# Ectopic fat in muscle and poor glycemic control are negatively associated with trabecular bone score in type 2 diabetes

**DOI:** 10.1016/j.clinsp.2024.100430

**Published:** 2024-07-10

**Authors:** Iana Mizumukai de Araújo, Carlos Ernesto Garrido Salmon, Francisco José Albuquerque de Paula

**Affiliations:** a Department of Internal Medicine, Faculdade de Medicina de Ribeirão Preto da Universidade de São Paulo (USP), Ribeirão Preto, SP, Brazil; b Department of Physics, Faculdade de Filosofia, Ciências e Letras de Ribeirão Preto da Universidade de São Paulo (USP), Ribeirão Preto, SP, Brazil

**Keywords:** Bone mineral Density, Magnetic resonance, Dual-energy X-Ray Absorptiometry, Intramuscular lipids

## Abstract

**Introduction:**

Type 2 Diabetes (T2D) is associated with fractures, despite preserved Bone Mineral Density (BMD). This study aimed to evaluate the relationship between BMD and trabecular bone score (TBS) with the reallocation of fat within muscle in individuals with eutrophy, obesity, and T2D.

**Methods:**

The subjects were divided into three groups: eutrophic controls paired by age and sex with the T2D group (*n* = 23), controls diagnosed with obesity paired by age, sex, and body mass index with the T2D group (*n* = 27), and the T2D group (*n* = 29). BMD and body fat percentage were determined using dual-energy X-Ray absorptiometry. TBS was determined using TBS iNsight software. Intra and extramyocellular lipids in the soleus were measured using proton magnetic resonance spectroscopy.

**Results:**

TBS was lower in the T2D group than in the other two groups. Glycated hemoglobin (A1c) was negatively associated with TBS. Body fat percentage was negatively associated with TBS and Total Hip (TH) BMD. TH BMD was positively associated with intramuscular lipids. A trend of negative association was observed between intramuscular lipids and TBS.

**Conclusion:**

This study showed for the first time that the reallocation of lipids within muscle has a negative association with TBS. Moreover, these results are consistent with previous studies showing a negative association between a parameter related to insulin resistance (intramuscular lipids) and TBS.

## Highlights

• Relationship between bone mineral density and trabecular bone score.
• Body fat percentage has negative relation with trabecular bone score.
• Reallocation of lipids within muscle has a negative relationship with trabecular bone score.

## Introduction

Osteoporosis and Type 2 Diabetes (T2D) are major contemporaneous human health disorders strongly associated with aging.^1^ Furthermore, obesity, another trait of modern society, has a divergent link to T2D and bone mass. Although obesity is considered a major risk factor for T2D, it has a positive relationship with bone mass.^2^ Previous studies have unanimously shown that body weight has a beneficial effect on bone mass.^3^ More recently, it has been recognized that obesity does not protect against fracture occurrence.^4^, ^5^, ^6^ In all probability, obesity impairs bone quality, not quantity. In the same vein, T2D is one of the diseases associated with a higher increased risk of fractures, although individuals with T2D show normal or increased Bone Mineral Density (BMD).^7^^,^^8^ Trabecular Bone Score (TBS) estimates bone texture based on pixel gray-level variations in previously obtained exams of Lumbar Spine (LS) densitometry. Several studies have indicated that TBS is especially useful in detecting bone impairment in obesity and T2D.^9^

Muscle and white adipose tissue dysfunction contribute differently to the emergence of T2D and bone fragility. The amount and distribution of White Adipose Tissue (WAT) are important determinants of metabolic homeostasis. Particularly, the deposition of WAT into Visceral Adipose Tissue (VAT) elicits insulin resistance and redistribution of lipids to other tissues. For instance, insulin resistance and reallocation of lipids are directly involved in the occurrence of steatohepatitis, impairment in muscle uptake of glucose, and cardiovascular disorders. However, a recent study showed that VAT does not have a negative relationship with bone mass.^10^ Moreover, obesity and congenital lipodystrophy, respectively common and rare disorders in commonality have increased insulin resistance as well as enhanced bone mass.^11^ Although bone has proper adipose tissue (Marrow Adipose Tissue [MAT]), MAT is not a place for increased fat storage in T2D. Patsch et al. and de Araújo et al. showed that the MAT amount in subjects with T2D is similar to that observed in controls.^12^^,^^13^ Additionally, Patsch et al. found that the lipids profile of MAT may determine fracture susceptibility. They observed that increased saturated and decreased unsaturated lipids in MAT are related to fragility fractures and normoglycemic and T2D.^12^ However, muscles play a key role in bone development and maintenance.^14^ The bone-muscle interaction encompasses the obvious mechanical effect and the biochemical crosstalk between myocytes and skeletal cells.^15^ T2D potentially affects both effects of muscle on bone. Previous studies have shown impairment in muscle performance and alterations in myokine production.^16^ Muscle accumulation of lipids within the extra- and intracellular microenvironments may, at least in part, be involved in this process.

Body weight has a positive effect on bone. However, there are contradictory results concerning the importance of adipose tissue and muscle mass in BMD determination. The hormonal profile of obesity that encompasses molecules with positive (estrogen, androgen, and insulin), negative (proinflammatory cytokines, hypovitaminosis D), or both positive and negative (leptin and adiponectin) effects on bone is an important piece of the complex relationship between adipose tissue and bone.^17^^,^^18^

Recently, it has been shown that several parameters that directly or indirectly reflect insulin resistance (HOMA-IR, VAT, and intrahepatic lipids) have no relationship with bone quantity but have a negative association with TBS.^10^ In the previous study, two key factors were not analyzed, the quantity of lipids in muscle and the percentage of body fat. These factors could help elucidate the relationship between body fat mass and bone.

This study aimed to evaluate the relationship between BMD and TBS and the reallocation of fat within the muscle in individuals with eutrophy, obesity, and T2D. Additionally, it aimed to assess the association between body fat percentage and BMD with TBS.

## Materials and methods

The STROBE checklist was followed for reporting this study.

### Subjects

A total of 79 individuals were included in this study. They were divided into 3 groups: eutrophic controls (C group, *n* = 23, 16 women and 7 men) paired by age and sex with the T2D group, controls diagnosed with obesity (P group, *n* = 27, 18 women and 9 men) paired by age, sex, and Body Mass Index (BMI) with the T2D group, and subjects with T2D (T2D group, *n* = 29, 17 women and 12 men). The experimental protocol was approved by the Institutional Review Board of the University Hospital of the Ribeirão Preto Medical School, USP (#52563116.7.00005414). The authors used posters and television press to recruit volunteers to participate in the study. Written informed consent was obtained from all subjects after receiving information about the risks and any eventual discomfort during the examinations.

The inclusion criteria for T2D group was had been diagnosed with T2D at least five years before the study. The diagnoses of T2D were according to the criteria of the Brazilian Diabetes Association and the American Diabetes Association. Exclusion criteria for all three groups included: pregnancy, early menopause, smoking and alcoholism, presence of a chronic disease known to affect bone metabolism, abnormal thyroid functioning, hypothalamic or pituitary disorders, glucocorticoid or osteoporosis therapy, nephropathy, proliferative retinopathy, and clinical neuropathy. All subjects in the T2D group had been diagnosed with T2D at least five years before the study and were in use of metformin.

### Laboratory analysis

Blood samples were obtained after 8-h overnight fasting. The biochemical assessment of total glucose, albumin, and creatinine was performed using an automatic biochemical analyzer (CT 600i, Wiener Lab Group, Rosario, Argentina). Glycated hemoglobin (A1c) levels were measured using High-Performance Liquid Chromatography (HPLC) (D10-Hemoglobin A1C Testing System, Bio Rad).

### Dual-energy X-Ray absorptiometry (DXA)

BMD in the LS (L1–L4), Total Hip (TH), and Femoral Neck (FN) and total body fat percentage were determined using DXA (Hologic Discovery Wi, QDR series, Waltham, MA, USA). TBS assessment was performed using TBS iNsight version 2.2 (Medimaps, Geneva, Switzerland). TBS measurements were performed in subjects with BMI values of 15–37 kg/m^2^ based on the manufacturer's recommendations. TBS was not measured in 5 subjects in the P group and 2 subjects in the T2D group due to the lack of BMI criteria or problems with data management.

### Proton magnetic resonance spectroscopy

The subjects were positioned feet-first in the prone position in the magnet bore of a 3T MRI scanner (Philips, Achieva). An XL torso coil was positioned over the proximal tibia. Axial T2-weighted fast spin-echo acquisition was used as a reference for the spectroscopy voxel placement (TE = 11 ms, TR = 400 ms, gap = 1.0 mm, slice thickness = 4 mm, and FOV = 22 cm). A single voxel of 20 × 20 × 49 cm^3^ was positioned at the soleus muscle. A point-resolved spectroscopy acquisition technique was applied using the following parameters: TR = 2400 ms, TE = 36 ms, 48 acquisitions with water suppression, and 8 acquisitions without water suppression. Extra- and Intramyocellular Lipids (EMCL and IMCL) were quantified from the spectra using LCModel software (version 6.1, http://www.s-provencher.com/pages/lcmodel.shtml). IMCL and EMCL estimates were automatically scaled to unsuppressed water peak and later expressed as IMCL-to-EMCL ratio. IMCL-to-EMCL ratios were not measured in 1 subject in the C group, 7 subjects in the P group, and 5 subjects in the T2D group due to the low signal-to-noise ratio of the spectra associated with the patient's movement.

### Statistical analysis

Statistical differences in the clinical variables were verified among the groups using a simple variance test (one-way analysis of variance) followed by Tukey's post-test using the R Core Team (Vienna, Austria, 2016). Linear regressions were applied using two models to determine the association among the evaluated parameters. The first linear regression model was a simple one disregarding any confounding factors (Model 1), and the second linear regression model was adjusted by age, A1c, and BMI (Model 2). The gender difference between the groups was verified using a chi-square test. Statistical significance was set at 0.05 for all statistical tests.

## Results

The C group was paired by age, sex, and height, and the P group was paired by age, sex, height, and BMI with the T2D group. Table 1 shows the anthropometric characteristics and biochemical evaluation of the three groups. The age ranges were 36–70 years in the C group, 33–71 years in the P group, and 39–66 years in the T2D group. The weight and BMI were lower in the C group than in the P and T2D groups. Serum creatinine and albumin levels were similar in the three groups. All subjects had these parameters within normal ranges. Glucose levels and A1c were higher in the T2D group than in the other two groups (Table 1).

**Table 1:** Clinical characteristics of the three groups: Control (C), Paired (P), and Type 2 Diabetes (T2D) groups, expressed as mean ± standard deviation.

	C (*n* = 23)	P (*n* = 27)	T2D (*n* = 29)
Age (years)	49 ± 11	51 ± 10	55 ± 8
Height (m)	1.66 ± 0.08	1.66 ± 0.08	1.64 ± 0.10
Weight (kg)	62.9 ± 7.5^a^	85.2 ± 15.1	83.5 ± 14.1
BMI (kg/m²)	22.9 ± 1.7^a^	30.6 ± 5.3	30.9 ± 4.6
Glucose level (mg/dL)	88 ± 7	92 ± 11	158 ± 61^b^
A1c (%)	5.2 ± 0.5	5.4 ± 0.3	8.3 ± 2.0^b^
Creatinine (µmol/L)	70.7 ± 17.7	70.7 ± 17.7	70.7 ± 17.7
Albumin (mmol/L)	0.60 ± 0.03	0.60 ± 0.03	0.60 ± 0.03
Vitamin D	29 ± 10	24 ± 6^f^	23 ± 8
LS BMD (g/cm²)	0.975 ± 0.089	1.032 ± 0.130	1.048 ± 0.152
FN BMD (g/cm²)	0.739 ± 0.092	0.844 ± 0.135^c^	0.865 ± 0.140^d^
TH BMD (g/cm²)	0.844 ± 0.099	0.940 ± 0.142^c^	0.995 ± 0.132^d^
Body fat percentage (%)	36.0 ± 5.7	41.7 ± 6.6^c^^,^^e^	36.7 ± 7.3
L1–L4 TBS	1.40 ± 0.08 f	1.34 ± 0.12^e^	1.26 ± 0.14
IMCL/EMCL	25.5 ± 20.3	30.7 ± 15.2	42.0 ± 20.4^d^

a Significant differences: (P and T2D) > C

b T2D > (P and C)

c P > C

d T2D > C

e P > T2D

f C > T2D BMI: body mass index; BMD: bone mineral density; TH: total hip; FN: femoral neck; LS: lumbar spine (L1–L4); TBS: trabecular bone score; A1c: glycated hemoglobin; IMCL/EMCL: intramyocellular/extramyocellular lipid ratio.

Table 1 shows the results of bone densitometry. LS BMD values were similar between the groups. TH and FN BMD values were lower in the C group than in the other two groups. In contrast, the L1–L4 TBS values were lower in the T2D group than in the other two groups. Furthermore, the mean value of TBS was lower in the P group than in the C group.

Body fat percentage measured by DXA was higher in the P group than in the other two groups. The IMCL/EMCL ratio was significantly higher in the T2D group (73 %) than in the C group, but the IMCL/EMCL ratio in the P group was similar to that in the other two groups (Table 1).

**Fig. 1:**
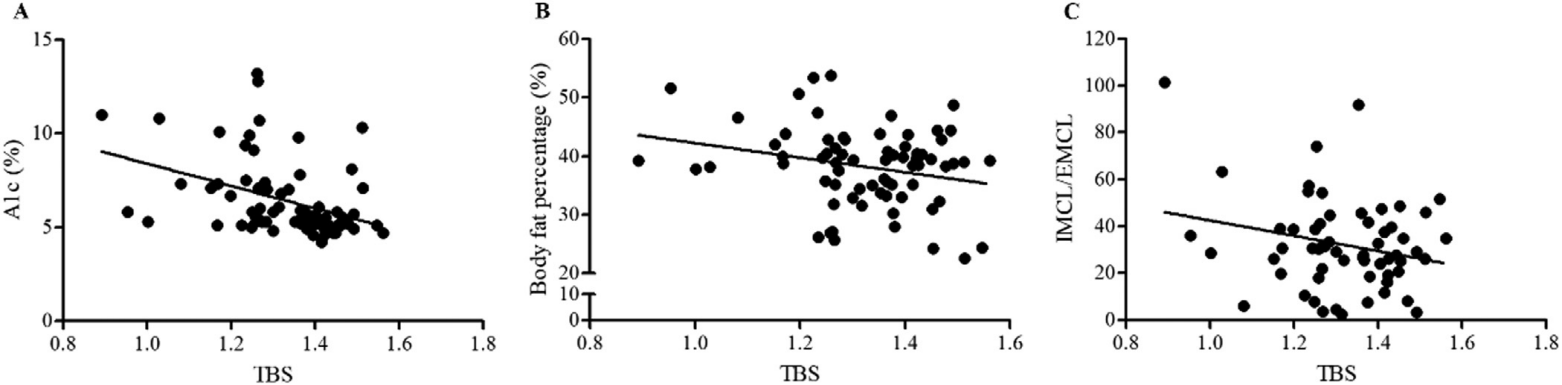
Associations between (A) glycated hemoglobin (A1c) and Trabecular Bone Score (TBS), (B) body fat percentage and TBS, and (C) Intra/Extramyocellular Lipid (IMCL/EMCL) ratio and TBS.

No association was observed between A1c and BMD measured in the three body regions (LS, TH, and FN), considering all individuals from the three groups. However, A1c was negatively associated with TBS (Fig. 1 A). Body fat percentage was negatively associated with TH BMD in Model 2 (p = 0.002). Additionally, body fat percentage was negatively associated with TBS in Model 1 (estimate = -0.05; p = 0.03) (Fig. 1 B).

**Fig. 2:**
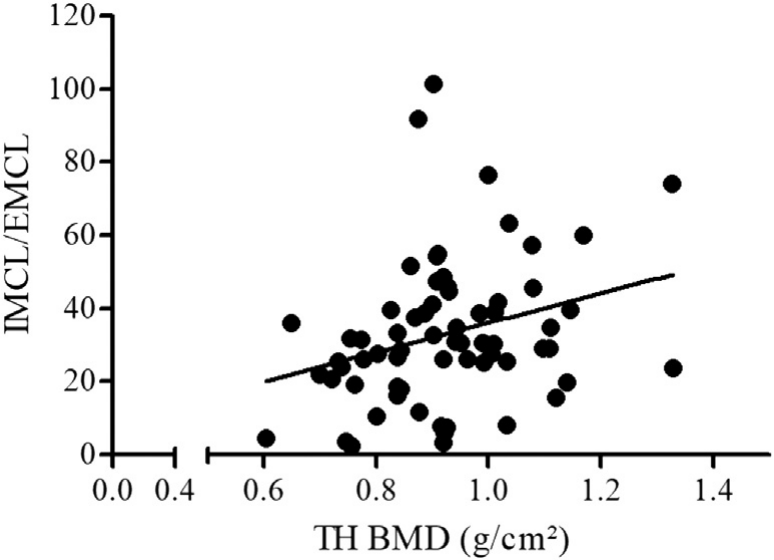
Association between the Intra/Extramyocellular Lipid (IMCL/EMCL) ratio and Total Hip Bone Mineral Density (TH BMD).

A positive association was observed between TH BMD and the IMCL/EMCL ratio (Table 2, Fig. 2), whereas no relationship was observed between the IMC/EMC ratio and LS and FN BMD. These findings suggest that insulin resistance does not have a negative effect on bone mass. Additionally, the analysis of the link between TBS and muscle lipids showed a trend for a negative association between IMCL/EMCL and TBS (Model 1) (Fig. 1 C).

**Table 2:** Association analysis including all the subjects of the study considering two models: model 1 is a simple linear model, and model 2 is a multiple linear regression model, adjusted by BMI, A1c, and age.

Associations		Model 1			Model 2		
Dependent	Independent	Estimate	Standard error	p-value	Estimate	Standard error	p-value
TH BMD (g/cm²)	A1c (%)	0.01	0.008	0.06	0.01	0.008	0.16
FN BMD (g/cm²)	A1c (%)	0.01	0.007	0.09	0.01	0.007	0.14
LS BMD (g/cm²)	A1c (%)	0.006	0.007	0.4	0.003	0.008	0.66
L1–L4 TBS	A1c (%)	**-0.02**	**0.007**	**0.0006**	- 0.011	0.006	0.09
TH BMD (g/cm²)	Body fat percentage (%)	-0.0032	0.002	0.2	- 0.007	**0.0023**	**0.002**
FN BMD (g/cm²)	Body fat percentage (%)	0.0002	0.0023	0.9	-0.0026	0.0023	0.27
LS BMD (g/cm²)	Body fat percentage (%)	-0.0011	0.002	0.6	-0.0035	0.0025	0.15
L1–L4 TBS	Body fat percentage (%)	**-0.05**	**0.0023**	**0.03**	-0.0004	0.0023	0.87
TH BMD (g/cm²)	IMCL/EMCL	**0.002**	**0.001**	**0.018**	0.001	0.001	0.14
FN BMD (g/cm²)	IMCL/EMCL	0.001	0.001	0.11	0.001	0.001	0.51
LS BMD (g/cm²)	IMCL/EMCL	0.001	0.001	0.54	0.000	0.001	0.95
L1–L4 TBS	IMCL/EMCL	-0.002	0.001	0.07	0.000	0.001	0.72

p < 0.05 indicates statistical significance. BMD, Bone Mineral Density; TH, Total Hip; FN, Femoral Neck; LS, Lumbar Spine (L1–L4); TBS, Trabecular Bone Score; A1c, glycated hemoglobin; IMCL/EMCL, Intramyocellular/Extramyocellular Lipid ratio.

## Discussion

Obesity and insulin resistance are two intimate disorders that negatively affect almost all tissues and systems, suppressing health, eliciting functional alterations in classical and nonclassical insulin-dependent cells, and driving the resurgence of several diseases. T2D, arterial hypertension, cardiovascular diseases, nonalcohol-related fatty liver disease, and cancer are strongly associated with obesity and insulin resistance. In obesity, T2D, and rare conditions of severe insulin resistance, bone mass is preserved or even increased.^11^^,^^13^ Despite this, individuals with obesity are not protected from fractures, and T2D increases the risk of fractures.^18^^,^^19^ A recent study has reported that insulin resistance and parameters strongly associated with insulin resistance (e.g., VAT and intrahepatic lipids) have a negative relationship with TBS, but not with BMD.^10^ This study adds to this line of investigation, showing that TBS can capture a spectrum of impairment in bone texture from obesity to T2D. Moreover, the results showed that the ectopic storage of lipids in muscle has a positive association with TH BMD and a neutral association with LS and FN BMD. These results reinforce previous suggestions that insulin resistance does not exert a detrimental effect on bone quantity.^10^ A trend of negative association was observed between TBS and the IMCL/EMCL ratio, a parameter linked to insulin resistance and decreased muscle uptake of glucose. Therefore, the study results reinforce the idea that insulin resistance does not negatively affect bone mass and support the use of the TBS tool to evaluate fracture risk in T2D.

Obesity is the most important modifiable risk factor for T2D development. Usually, the installation of T2D in individuals with obesity heightens the chance of comorbidities associated with obesity, such as cardiovascular disorders and steatohepatitis.^20^ T2D is associated with high bone mass preservation. Moreover, rapid and intense weight loss creates conditions for T2D remission^21^ but provokes bone loss.^22^ As such, bone mass has a unique relationship with obesity and T2D, in view that obesity has a positive effect on bone mass, and T2D does not impair this positive influence. The study results showed that BMD was higher in the P and T2D groups than in the control group. It is necessary to highlight that BMD was slightly higher in the T2D group than in the P group in all regions. Thus, these results are consistent with those reported in a previous study by de Araújo et al. (2018), which showed that bone mass was slightly higher in subjects with T2D than in subjects with overweight and obesity.^13^ These results suggest that bone mass estimation by DXA does not differentiate fracture risk between obesity and T2D.

A previous study showed that TBS detects differences in bone impairment between individuals with obesity diagnosed with primary obesity and those with Cushing disease.^23^ Additionally, it was observed that TBS captures differences in the impairment of bone texture in obesity and T2D. Individuals with obesity showed lower TBS than the control group, but individuals diagnosed with T2D showed lower TBS than the control group and those with obesity and overweight. Currently, there is no ideal exam for the identification of individuals with T2D that has high fracture risk and would benefit from therapy to reduce fracture risk. However, the addition of TBS to the fracture risk assessment algorithm has been suggested as a tool to circumvent the limitations of currently available exams and ameliorate the evaluation of subjects with T2D for osteoporosis treatment. These findings prove that TBS is useful for detecting the spectrum of bone alterations in obesity and T2D.

The study results showed that poor metabolic control, as estimated by A1c, has a negative association with TBS, but not with BMD. The mechanisms that determine bone deterioration in T2D remain to be elucidated. In the last two decades, the notion that complex metabolic alterations in diabetes mellitus involving carbohydrates, lipids, and proteins exert diverse toxic effects on bone cells and affect bone structure has increased. The enrichment of collagen cross-links with advanced glycation end products (e.g., glucosepane and pentosidine) is a natural occurrence in the hyperglycemic environment. It seems to hamper the mineralization process of bone and the potential for repairing the skeleton.^24^ Additionally, the bone remodeling rate is reduced in diabetes mellitus, indicating that bone formation and resorption are reduced.^25^ Evidence suggests that hyperglycemia has a direct effect on osteoblast lineage cells, suppressing differentiation and maturation.^26^ In parallel, hyperglycemia may reduce osteoclast activation by decreasing RANKL signaling, which results in the deactivation of bone resorption.^27^ However, how T2D affects the analysis of bone texture by TBS is unclear. In HRPqCT exams, it is described that T2D is associated with alterations in the cortical compartment of bone, exhibiting increased cortical porosity, lower cortical volumetric BMD, and smaller cross-sectional area. The association between TBS and HRPqCT parameters in T2D remains to be evaluated.

Body fat mass percentage had no relationship with BMD, but a negative association with LS TBS was observed. Also, the P group had a higher body fat percentage than the other two groups. It probably reflects that it is not the quantity but rather the distribution of body fat that is related to the development of type 2 diabetes, as pointed out previously.^28^ The complex relationship between bone and adipose tissue in obesity and T2D can easily be highlighted by different angles of analysis. For instance, obesity and T2D are two classical examples of vitamin D deficiency, but both are associated with high bone mass. Despite vitamin D deficiency, T2D is not associated with hypersecretion of parathyroid hormone.^29^ Obesity and T2D are distinguished by high and low serum levels of leptin and adiponectin, respectively. However, these hormones have positive (directly) and negative (via central action) effects on bone.^30^

This study has some limitations. This was a cross-sectional study and included a small sample size. However, subjects were divided into 3 groups, which allowed a comparison of individuals with T2D not only with normoglycemic individuals showing body weight in the normal range but also with a normoglycemic group including individuals with overweight and obesity. Moreover, the content of muscle lipids was estimated using proton magnetic resonance spectroscopy, an appropriate and noninvasive method to determine fat within different tissues.

In conclusion, body fat percentage, ectopic lipid amount in muscle, and poor glycemic control, measured by A1c, were negatively associated with TBS. These results indicate that increased body fat and ectopic fat and poor glycemic control are related to poorer bone quality. This study reinforces the importance of adding TBS to evaluate T2D bone health and the beneficial effects of body fat loss on bone quality.

## Ethical approval

The experimental protocol was approved by the Institutional Review Board of the University Hospital of the Ribeirão Preto Medical School ‒ USP (#52563116.7.00005414). Written informed consent was obtained from all subjects after receiving information about the risks and any eventual discomfort during the examinations. The procedures were performed in accordance with the Declaration of Helsinki.

## Authors’ contributions

I.M.A., C.E.G.S, and F.J.A.P designed the experimental protocol, performed research, analyzed data and wrote the manuscript, and revised the manuscript.

## Declaration of competing interest

The authors declare no conflicts of interest.

## References

[1] Cao JJ. (2011). Effects of obesity on bone metabolism. J Orthop Surg Res.

[2] Viggers R, Al-Mashhadi Z, Fuglsang-Nielsen R, Gregersen S, Starup-Linde J. (2020). The impact of exercise on bone health in type 2 diabetes mellitus ‒ A systematic review. Curr Osteoporos Rep.

[3] Reid IR. (2010). Fat and bone. Arch Biochem Biophys.

[4] Premaor MO, Pilbrow L, Tonkin C, Parker RA, Compston J. (2010). Obesity and fractures in postmenopausal women. J Bone Miner Res.

[5] Compston JE, Watts NB, Chapurlat R, Cooper C, Boonen S, Greenspan S (2016). Obesity is not protective against fracture in postmenopausal women: GLOW MD s for the GLOW investigators. Am J Med.

[6] Compston J. (2013). Obesity and fractures. Jt Bone Spine.

[7] Sheu A, Greenfield JR, White CP, Center JR. (2022). Assessment and treatment of osteoporosis and fractures in type 2 diabetes. Trends Endocrinol Metab.

[8] Napoli N, Chandran M, Pierroz DD, Abrahamsen B, Schwartz AV, Ferrari SL (2017). IOF Bone and Diabetes Working Group. Mechanisms of diabetes mellitus-induced bone fragility. Nat Rev Endocrinol.

[9] Li G, Prior JC, Leslie WD, Thabane L, Papaioannou A, Josse RG (2019). Frailty and risk of fractures in patients with type 2 diabetes. Diabetes Care.

[10] de Araújo IM, Parreiras-e-Silva LT, Carvalho AL, Elias J, Salmon CEG, de Paula FJA. (2020). Insulin resistance negatively affects bone quality not quantity: the relationship between bone and adipose tissue. Osteoporos Int.

[11] Lima JG, Nobrega LHC, Lima NN, dos Santos MCF, Baracho MFP, Winzenrieth R (2017). Normal bone density and trabecular bone score, but high serum sclerostin in congenital generalized lipodystrophy. Bone.

[12] Patsch JM, Li X, Baum T, Yap SP, Karampinos DC, Schwartz AV, Link TM. (2013). Bone marrow fat composition as a novel imaging biomarker in postmenopausal women with prevalent fragility fractures. J Bone Miner Res.

[13] De Araújo IM, Salmon CEG, Nahas AK, Nogueira-Barbosa MH, Elias J, De Paula FJA. (2017). Marrow adipose tissue spectrum in obesity and type 2 diabetes mellitus. Eur J Endocrinol.

[14] Bonewald L. (2019). Use it or lose it to age: a review of bone and muscle communication. Bone.

[15] Brotto M, Bonewald L. (2015). LBone and muscle: interactions beyond mechanical. Bone.

[16] Garneau L, Aguer C. (2019). Role of myokines in the development of skeletal muscle insulin resistance and related metabolic defects in type 2 diabetes. Diabetes Metab.

[17] De Paula FJA, Rosen CJ. (2017). Structure and function of bone marrow adipocytes. Compr Physiol.

[18] Compston JE, Flahive J, Hooven FH, Anderson FA, Adachi JD, Boonen S (2014). Obesity, health-care utilization, and health-related quality of life after fracture in postmenopausal women: global longitudinal study of osteoporosis in women (GLOW). Calcif Tissue Int.

[19] de Araújo IM, Moreira MLM, de Paula FJA. (2022). Diabetes and bone. Arch Endocrinol Metab..

[20] Després J-P, Lemieux I. (2006). Abdominal obesity and metabolic syndrome. Nature.

[21] Lean ME, Leslie WS, Barnes AC, Brosnahan N, Thom G, McCombie L (2018). Primary care-led weight management for remission of type 2 diabetes (DiRECT): An open-label, cluster-randomised trial. Lancet.

[22] Alencar M, De Araújo IM, Parreiras-e-Silva LT, Nogueira-Barbosa MH, Salgado W, Elias J (2021). Hashtag bone: detrimental effects on bone contrast with metabolic benefits one and five years after Roux-en-Y gastric bypass. Braz J Med Biol Res.

[23] Batista SL, de Araújo IM, Carvalho AL, Alencar MAVSD, Nahas AK, Elias J (2019). Beyond the metabolic syndrome: visceral and marrow adipose tissues impair bone quantity and quality in Cushing's disease. PLoS One.

[24] Ogawa N, Yamaguchi T, Yano S, Yamauchi M, Yamamoto M, Sugimoto T. (2007). The combination of high glucose and advanced glycation end-products (AGEs) inhibits the mineralization of osteoblastic MC3T3-E1 cells through glucose-induced increase in the receptor for AGEs. Horm Metab Res.

[25] Vavanikunnel J, Sewing L, Triantafyllidou M, Steighardt A, Baumann S, Egger A (2022). Determinants of low bone turnover in Type 2 diabetes-the role of PTH. Calcif Tissue Int.

[26] Botolin S, McCabe LR. (2006). Chronic hyperglycemia modulates osteoblast gene expression through osmotic and non-osmotic pathways. J Cell Biochem.

[27] Picke AK, Campbell G, Napoli N, Hofbauer LC, Rauner M. (2019). Update on the impact of type 2 diabetes mellitus on bone metabolism and material properties. Endocr Connect.

[28] Agrawal S, Klarqvist MDR, Diamant N., Stanley T.L., Ellinor P.T. (2023). BMI-adjusted adipose tissue volumes exhibit depot-specific and divergent associations with cardiometabolic diseases. Nat Commun.

[29] Paula FJA, Lanna CMM, Shuhama T, Foss MC. (2001). Effect of metabolic control on parathyroid hormone secretion in diabetic patients. Braz J Med Biol Res.

[30] Ducy P, Amling M, Takeda S, Priemel M, Schilling AF, Beil FT (2000). Leptin inhibits bone formation through a hypothalamic relay: a central control of bone mass. Cell.

